# Difference Analysis on Virulence Genes, Biofilms and Antimicrobial Susceptibility of *Escherichia coli* from Clinical and Subclinical Bovine Mastitis

**DOI:** 10.3390/vetsci12020132

**Published:** 2025-02-06

**Authors:** Jiakun Zuo, Zhaoyang Lv, Liyan Lian, Zihao Wu, Shaodong Fu, Haiyang Zhang, Jing Wu, Zihao Pan, Yong Yu, Wei Chen, Wei Jiang, Huifang Yin, Zhaoguo Chen, Yunpeng Yi, Xiangan Han, Jinfeng Miao

**Affiliations:** 1 College of Veterinary Medicine, Nanjing Agricultural University, Nanjing 210095, China; 2 Shanghai Veterinary Research Institute, The Chinese Academy of Agricultural Sciences (CAAS), 518 Ziyue Road, Shanghai 200241, China; 3 College of Animal Science and Technology, Tarim University, Alar 843300, China; 4 Engineering Research Center for the Prevention and Control of Animal Original Zoonosis, Fujian Province, College of Life Science, Longyan University, Longyan 364012, China; 5 Shandong Provincial Animal and Poultry Green Health Products Creation Engineering Laboratory, Institute of Poultry Science, Shandong Academy of Agricultural Science, Jinan 250100, China

**Keywords:** *Escherichia coli*, bovine mastitis, MLST, biofilm, virulence gene, antimicrobial resistance

## Abstract

*Escherichia coli* (*E. coli*) has the ability to induce clinical and subclinical mastitis in dairy cows, causing a huge loss for the dairy industry. In this study, 51 subclinical mastitis isolates and 36 clinical mastitis isolates from eight provinces of China between 2019 and 2021 were used to investigate the differences in their biological characteristics. The results showed that B1 (52.9%) and A (39.1%) were the predominant phylogroups; R1 (50.6%) was the predominant lipopolysaccharide (LPS) core type; and 44 STs (ST10 and ST58 were the most sequence-prevalent STs) and 2 new STs (ST14828 and ST14829) were identified; however, no significant difference was observed between the clinical and subclinical group strains. To compare the virulence gene differences between the clinical and subclinical mastitis-related isolates, 18 common virulence genes (including *afaE*, *eaeA*, *papC*, *saa*, *sfa*, *ompA*, *aer*, *irp2*, *iucD*, *escV*, *sepD*, *east1*, *estB*, *stx2e*, *CNF1*, *cba*, *hlyA* and *traT*) were determined using the PCR method. The results showed that the detection rates of *traT*, *irp2* and *iucD* in clinical mastitis isolates were significantly higher than those in subclinical mastitis isolates (*p* ˂ 0.05). Meanwhile, subclinical-group *E. coli* had stronger biofilm formation abilities than the clinical group (*p* < 0.05) in 78 (89.7%) mastitis-related *E. coli* that could form biofilms. Furthermore, 87 mastitis-related *E. coli* showed severe resistance against tetracycline (37.9%), ampicillin (36.8%), streptomycin (34.5%) and cotrimoxazole (28.7%); their most prevalent resistance genes were *blaCTX-M* (33.3%), *tetA* (27.6%), *sul2* (18.4%) and *strB* (28.7%). It was noteworthy that the clinical-group strains had a higher resistance against ampicillin and possessed higher amounts of the resistance gene *blaCTX-M* (*p* < 0.05) compared to the subclinical group. This study aims to provide references for preventing the *E. coli* isolates from inducing different types of mastitis.

## Simple Summary

Bovine mastitis is the most common disease in the dairy industry worldwide and results in serious damage to both animal welfare and economic benefit. *Escherichia coli* (*E. coli*) is one of the important pathogens that could induce clinical and subclinical mastitis in dairy cows. Bovine mastitis-related *E. coli* show considerable genetic diversity, and the differences between the clinical and subclinical mastitis-related strains are unclear. This study compared the two groups of strains from the aspects of molecular subtyping, virulence genes, biofilms and resistance. This study aimed to investigate the molecular epidemiological characteristics of mastitis-related *E. coli* and to provide references for their prevention.

## 1. Introduction

Bovine mastitis is considered to be a devastating disease for the dairy industry throughout the world. It is responsible for great losses for dairy producers and the milk processing industry as a result of reduced milk production, the alteration of milk composition, treatment costs and even severe systemic infection [1]. Many factors contribute to the development of mastitis, including hosts, pathogens and environmental factors; bacterial infection is the most crucial one [2]. Most bovine mastitis pathogens are classified as contagious and environmental pathogens depending on their distribution in their natural habitat and on their mode of transmission from their natural habitat to the mammary glands of cows [3]. Among the contagious pathogens, *Staphylococcus aureus*, *Streptococcus dysgalactiae* and *Streptococcus agalactiae* are predominant, while *Escherichia coli* (*E. coli*), *Klebsiella pneumoniae*, coagulase-negative staphylococci (CNS) and *Streptococcus uberis* are the most common environmental pathogens [4,5]. Compared with contagious pathogens, environmental pathogens are more difficult to control because they exist in the environment of dairy cows and can transmit to the mammary glands at any time during the cows’ daily activities, such as lying down or milking.

According to the clinical symptoms, mastitis can be also classified into clinical and subclinical forms. Clinical mastitis can be identified easily based on visible symptoms of the udders, such as redness, warmth, swelling, pain, fever and abnormal lactation; no visible symptoms are observed in cows with subclinical mastitis [6]. Although the level of inflammation of clinical mastitis is more serious than that of subclinical mastitis, the economic loss induced by subclinical mastitis cannot be overlooked due to its higher prevalence, longer duration and the damage it causes in relation to milk quality and production [4].

Due to its high frequency, serious resistance and huge economic loss, mastitis caused by *E. coli* is a global concern in the dairy farming industry. Mastitis caused by *E. coli* occurs at a high frequency in dairy farming, and almost 80% of mastitis found in mammary infections during the dry period is caused by *E. coli* [7]. *E. coli* is often related to rapid-onset, acute mastitis with severe symptoms, and it is also observed in subclinical mastitis [8]. In the past, most studies focused on clinical mastitis-related *E. coli*, and the differences between the clinical and subclinical strains were unclear [1].

Antibiotics are still the most common method of treatment for coliform mastitis. However, due to their extensive use, the emergence of antimicrobial-resistant bacteria has posed a challenge to both the prevention of bovine mastitis and public health throughout the food chain. Antibiotic-resistant *E. coli* in milk is a serious concern since this bacterial species has a great capacity to accumulate and transmit antibiotic resistance genes (ARGs) through horizontal gene transfer [9].

To learn more about the features of mastitis-related *E. coli* strains, in this study, 87 *E. coli* isolated from 51 samples of subclinical mastitis milk and 36 samples of clinical mastitis milk, respectively, in China between 2019 and 2021, were used as the main study objects, and their biological characteristics were determined. This study aims to provide references for studying the prevention of mastitis-related *E. coli.*

## 2. Materials and Methods

### 2.1. Bovine Mastitis-Related E. coli

**Figure 1:**
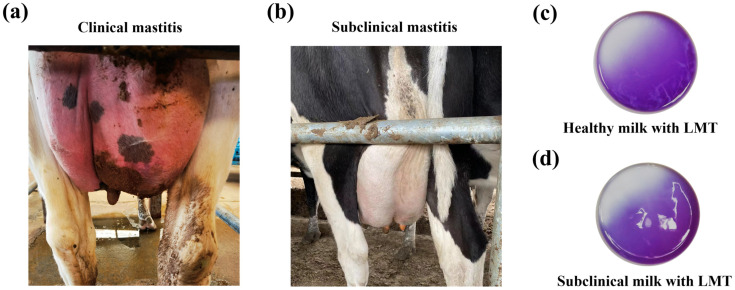
Bovine mastitis types. (**a**) A dairy cow suffering from clinical mastitis. The diagnosis of clinical mastitis is based on clinical signs, e.g., swelling of the udder, tenderness to touch, fever and depression. (**b**) A dairy cow suffering from subclinical mastitis. Usually, there are no obvious changes in the mammary gland of subclinical mastitis cases, e.g., lack of swelling and other clinical signs. CMT was performed using Lanzhou Mastitis Test (LMT) reagent to verify udder health/quarter milk quality. (**c**) Coagulum negative in the test cup after mixing healthy milk with the LMT reagent; (**d**) there was obvious coagulum formed after mixing the milk sample with the LMT reagent.

In this study, 87 *E. coli* strains were isolated from the milk samples of different dairy cows in China between 2019 and 2021, including 51 subclinical mastitis-related strains and 36 clinical mastitis-related strains. The diagnosis of clinical and subclinical bovine mastitis cases, as well as the milk collection, was performed as described previously [10]. Cows suffering with clinical mastitis were diagnosed based on clinical signs, e.g., the swelling of the udder, tenderness to touch, fever and depression (Figure 1a). Subclinical bovine mastitis cases were diagnosed using the California Mastitis Test (CTM) method and were tested using the diagnostic reagent Lanzhou Mastitis Test (LMT) (Figure 1b–d). The *E. coli* strains were isolated using MacConkey agar. The milk samples were cultivated onto MacConkey agar using sterile cotton swabs and were then incubated at 37 °C overnight. The lactose-positive colonies were picked into Luria–Bertani (LB) medium and were identified via the PCR amplification of the *phoA* gene with the primers phoA-F/phoA-R as well as 16S rRNA gene sequencing. All the primers used in this study are listed in Supplementary Table S1, and detailed information on the isolates is listed in Supplementary Table S2.

### 2.2. Extraction of Bacterial Genomic DNA

The *E. coli* strains isolated from cows with mastitis were streaked on LB agar plates and cultured at 37 °C overnight. A single colony of each strain was inoculated into 5 mL LB broth. The genomic DNA of the bacteria was extracted from a 1 mL overnight culture using a boiling-water extraction method, as detailed in a previous study [11].

### 2.3. Phylogenetic Analysis, Lipopolysaccharide (LPS) Core Types and Multilocus Sequence Typing (MLST) of Mastitis-Related E. coli

To analyze the strains and then verify whether there are any connections with, e.g., animals or case etiology, clinical *E. coli* isolates were phylotyped according to Clermont’s quadruplex PCR methods [12]. The genomic DNA of the *E. coli* isolates served as templates for the PCR reactions. The *E. coli* isolates were assigned to one of the eight phylogroups (A, B1, B2, C, D, E, F and clade I) by scoring the presence/absence of the genes in the order *arpA*/*chuA*/*yjaA*/*TspE4.C2* using a quadruplex PCR assay. All the primers used in this study are listed in Supplementary Table S1.

The LPS core types of the *E. coli* isolates were determined via the PCR amplification of specific genes of the R1, R2, R3, R4 and K12 oligosaccharides. The primer sequences and PCR reaction were performed as in a previous study [13].

The MLST of the isolates was performed using the standardized Achtman scheme by sequencing seven housekeeping genes (*adk*, *fumC*, *gyrB*, *icd*, *mdh*, *purA* and *recA*), as has been previously described [14]. Alleles and sequence type (ST) were assigned according to the MLST database (https://pubmlst.org, accessed on 11 November 2022). The whole genomes of strains with novel STs were obtained using next-generation sequencing (NGS) and were submitted to the enterobase website (https://enterobase.warwick.ac.uk/, accessed on 11 July 2023) for molecular typing. A phylogenetic tree was created according to the STs using the goeBURST algorithm from PHYLOViZ Online (https://online.phyloviz.net, accessed on 24 July 2023).

### 2.4. Distribution of Virulence Genes in Mastitis-Related E. coli

To analyze the distribution of virulence genes in mastitis-related *E. coli*, specific PCR primers (Supplementary Table S1) of genes related to adherence (*afaE*, *eaeA*, *papC*, *saa* and *sfa*), invasion factor (*ompA*), siderophores (*aer*, *irp2* and *iucD*), type III secretion system (T3SS) (*escV* and *sepD*) and toxins (*east1*, *estB*, *stx2e*, *CNF1*, *cba* and *hlyA*) and the serum resistance factor (*traT*) were synthesized by Sangon Biotech (China); they were used to amplify the common virulence genes in mastitis-derived *E.coli*. PCR reactions were performed according to the previously described methods.

### 2.5. Biofilm Formation

The bacterial biofilms of the *E. coli* isolates were measured in 96-well polyvinyl chloride (PVC) microplates using the crystal violet method as described previously with some modification [15]. Briefly, bacteria that were cultured to *OD*_600_ = 1.0 were diluted 1:10 in LB, and 200 μL of the dilution was inoculated into 96-well microplates and cultured at 25 °C for 24 h. Each strain was repeated for 6 wells. The culture of the wells was washed thrice with PBS and dried off at 60 °C for 30 min. The wells were stained using 200 μL of 0.1% crystal violet for 15 min at 37 °C, and the excess crystal violet was washed off with PBS. After being air-dried, 200 μL of 95 % ethanol was added to the wells to dissolve the crystal violet; the absorbance at 595 nm (*OD*_595_) was measured using a Synergy 2 microplate reader (BioTek, Winooski, VT, USA). Six wells with sterile LB were used as blank control.

The biofilm formation abilities of the strains were analyzed by the optical density of the sample (*OD*_sample_, *OD*_s_) differentiated by the critical OD (*OD*_control_, *OD*_c_). The *OD*_c_ value and objective criteria were calculated according to the methods of a previous study [16]. The *OD*_c_ value was calculated from the arithmetic mean of the absorbance of negative control wells with three times the SD. When *OD*_s_ ≤ *OD*_c_, the bacteria did not form biofilms; when *OD*_c_ < *OD*_s_ ≤ 2*OD*_c_, the bacteria had a weak ability to form biofilms; when 2*OD*_c_ < *OD*_s_ ≤ 4*OD*_c_, the bacteria had a moderate ability to form biofilms; and when *OD*_s_ > 4*OD*_c_, the bacterial ability to form biofilms was strong.

### 2.6. Antimicrobial Susceptibility

The antimicrobial susceptibility of mastitis-related *E. coli* to 12 antimicrobial agents was measured by the Kirby–Bauer disk diffusion method according to the Clinical and Laboratory Standards Institute guidelines [17]. In brief, bacteria with 0.5 MacFarland turbidities were coated on Mueller–Hinton agar; then, commercially available disks were attached to the surface of the agar. After being incubated at 37 °C for 16 h, the diameter of the bacteriostatic zone was measured to assess the drug susceptibility of the bacteria. The commercial disks used here included *β*-lactam antimicrobials (ampicillin, cefradine, ceftazidime, cefepime and amoxicillin/clavulanate potassium), aminoglycoside antimicrobials (kanamycin, spectinomycin, gentamicin and streptomycin), tetracycline antimicrobials (tetracycline), phenicol antimicrobials (chloramphenicol), sulfonamide antimicrobials (cotrimoxazole), quinolone antimicrobials (ciprofloxacin and enrofloxacin) and polypeptide antimicrobials (polymyxin B), which were purchased from Wenzhou Kangtai Biotech (Taizhou, China). The drug breakpoints were referenced to criteria published by the CLSI (2020).

### 2.7. Distribution of Common Antibiotic-Resistant Genes

Based on resistance gene data from the Antibiotic Resistance Genes Database (ARDB), specific primers for common tetracyline-resistance-related genes (*tetA*, *tetB* and *tetC*), *β*-lactamase genes (*blaCTX-M*, *blaSHV*, *blaTEM*, *blaOXA* and *blaKPC*), sulfonamide resistance genes (*sul1*, *sul2* and *sul3*) and aminoglycoside resistance genes (*aadA*, *strA* and *strB*) were used in this study; these are listed in Supplementary Table S2. The PCR reaction conditions were set as follows: 12.5 μL 2 × Es Taq MasterMix buffer (Cwbiotech, Taizhou, China), 1 μL of each primer (10 μM), 1 μL bacterial DNA and ddH_2_O up to the reaction volume. PCR amplification was carried out as follows: 4 min initial denaturation at 94 °C, 30 cycles of 40 s denaturation at 94 °C, 40 s annealing at primer-specific annealing temperature and 1 min extension at 72 °C followed by a 10 min final extension at 72 °C. The PCR products were identified using 1% agarose gel electrophoresis.

The subtypes of CTX-M were identified via the Sanger sequencing of the PCR production of CTX-M genes by Sangon Biotech (Shanghai, China). Then, the sequences were placed in the NCBI nucleotide database (https://blast.ncbi.nlm.nih.gov, accessed on 14 June 2024) to distinguish the subtypes.

### 2.8. Phylogenetic Analysis Tree

A phylogenetic tree of the 87 strains according to the seven housekeeping genes (*adk*, *fumC*, *gyrB*, *icd*, *mdh*, *purA* and *recA*) was created using neighbor-joining from the MEGA 11 software. The visualization, annotation and decoration of the trees were carried out utilizing tvBOT (https://www.chiplot.online/tvbot.html, accessed on 22 August 2024) [18].

### 2.9. Statistical Analysis

GraphPad Prism 9.0 was used to analyze and visualize the data statistically. Student’s *t*-test and Fisher’s exact test were used to analyze the data, and *p* values < 0.05 were used to indicate statistical significance.

## 3. Results

### 3.1. Molecular Subtyping of Clinical and Subclinical Mastitis-Related E. coli Strains

**Figure 2:**
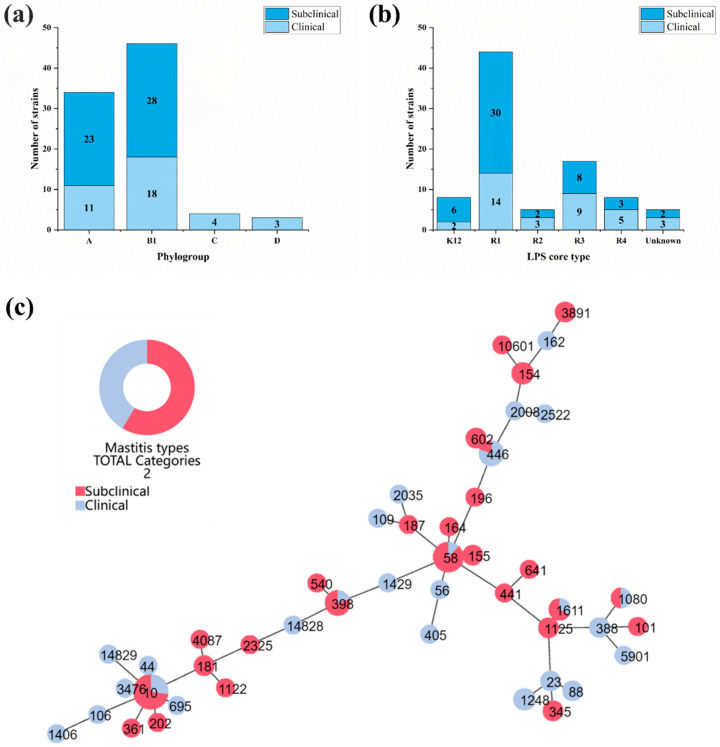
Molecular subtyping of bovine mastitis-related *E. coli.* (**a**) Distribution of five phylogroups in 87 mastitis-related *E coli* strains; (**b**) distribution of LPS core types of mastitis-related *E. coli* strains; (**c**) the eBRUST map of 87 mastitis-related *E. coli* strains according to their STs. STs with red color represent strains from 51 subclinical mastitis-related *E. coli*, and STs with blue color represent strains from 36 clinical mastitis-related *E. coli*.

According to the PCR results of the phylotyped genes (Supplementary Figure S1), four phylogroups were found in mastitis-related *E. coli*, including A, B1, C and D (Figure 2a). B1 (52.9%, 46/87) and A (39.1%, 34/87) were the predominant phylogroups, while a few strains belonging to groups C (4.6%, 4/87) and D (3.4%, 3/87) were found in clinical mastitis strains. The difference analysis showed that there was no significant difference among the four phylogroups between the clinical and subclinical group strains (*p* > 0.05).

According to the PCR results of the LPS core types, 82 strains were assigned to five different types, while 5 strains could not be assigned to any LPS core type due to the lack of amplification of the PCR assays. R1 was the most prevalent LPS core type of the isolates, accounting for 50.6% (44/87) followed by R3 (19.5%), K12 (9.2%), R4 (9.2%) and R2 (5.7%; Figure 2b). All the LPS core types were detected in both the subclinical and clinical mastitis-related isolates; there was no significant difference in the distributions of each LPS core type between the two groups (*p* > 0.05).

After analyzing the seven housekeeping genes, the 87 mastitis-related *E. coli* isolates were assigned to 44 known STs and 2 new STs (ST14828 and ST14829) (Supplementary Table S3). According to the phylogenetic tree of the MLST profiles (Figure 2c), the distribution of mastitis-related *E. coli* strains was highly heterogeneous. The most prevalent ST types were ST10 and ST58, with no significant difference between clinical and subclinical isolates.

### 3.2. Profiles of Virulence Genes

**Figure 3:**
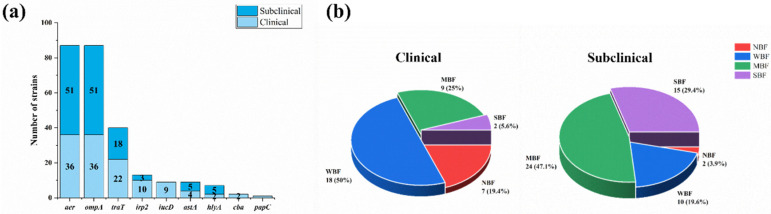
Virulence genes and biofilms of 87 mastitis-related *E. coli* strains. (**a**) Distribution of nine different virulence genes in 87 mastitis-related *E coli* strains. (**b**) Pie charts of distribution of strains with strong biofilm formation (SBF), moderate biofilm formation (MBF), weak biofilm formation (WBF) and no biofilm formation (NBF) for subclinical and clinical mastitis-related *E. coli* strains.

Among the eighteen detected virulence genes, nine were detected in mastitis-related *E. coli* isolates (Supplementary Figure S2). Other than *ompA* and *aer*, which were found in all the isolates, the detection rates of the other eight virulence genes were *traT* (46.0%), *irp2* (14.9%), *iucD* (10.3%), *astA* (10.3%), *hlyA* (8.0%), *cba* (2.3%) and *papC* (1.1%) (Figure 3a). According to Fisher’s exact test, the clinical mastitis isolates tended to possess more virulence genes than the subclinical strains in relation to *traT* (*p* < 0.05), *irp2* (*p* < 0.01) and *iucD* (*p* < 0.001).

### 3.3. Analysis of Biofilm Formation Abilities of Mastitis-Related E. coli

The *OD*_c_ value (0.34) was calculated using the arithmetic mean of the negative control (0.237) plus three times the SD (0.036). Most mastitis-related *E. coli* strains (89.7%, 78/87) could form biofilms, and the ratios for strong biofilm formation (SBF), moderate biofilm formation (MBF), weak biofilm formation (WBF) and no biofilm formation (NBF) were 19.5% (17/87), 37.9% (33/87), 32.2% (28/87) and 10.3% (9/87), respectively. Meanwhile, the constructions of biofilm-forming strains in the two groups are shown in Figure 3b; the subclinical group strains had stronger biofilm formation abilities than the clinical group (*p* < 0.05), which tended to have more strains with no biofilms (19.4%, 7/36) or a weak biofilm (50.0%, 18/36) than the subclinical group, which tended to have more strains with medium (47.1%, 24/51) and strong (29.4%, 15/51) biofilm formation capabilities than the clinical group.

### 3.4. Antimicrobial Susceptibility of Mastitis-Related E. coli

Mastitis-related *E. coli* exhibited resistance to 11 different antimicrobials except polymyxin B (Table 1). The mastitis-related *E. coli* strains mainly showed severe resistance to four classes of antimicrobials, including tetracyclines (37.9% to tetracycline) β-lactams (36.8% to ampicillin), aminoglycosides (34.5% to streptomycin) and sulfonamides (28.7% to cotrimoxazole); however, they were sensitive to amoxicillin plus clavulanic acid, enrofloxacin, cefepime and ceftazidime, with resistance rates of 1.1%, 4.6%, 5.7% and 6.9%, respectively. The isolates also showed resistance to the other antimicrobials, including gentamycin (17.2%), kanamycin (14.9%) and florfenicol (12.6%). The multiple-drug resistance (MDR—resistance to three or more classes of antimicrobials) of mastitis-related *E. coli* was further analyzed as shown in Table 2. The results showed that 36.8% (32/87) of the isolates exhibited multiple-drug resistance. The percentage of isolates resistant to 3, 4, 5 and 6 kinds of drugs was 11.5%, 8.0%, 6.9% and 3.4%, respectively.

To analyze the differences between clinical and subclinical mastitis, the antimicrobial susceptibilities and the MDR strains of the two groups were analyzed via the chi-square test. Overall, the resistance tendencies of the two groups were similar to most kinds of antimicrobials and MDR distributions, apart from those of ampicillin. There was a significant difference in the drug resistance of ampicillin between subclinical and clinical mastitis isolates; the clinical strains exhibited a higher resistance to ampicillin than the subclinical isolates (*p* < 0.05).

### 3.5. Resistance Genes

**Figure 4:**
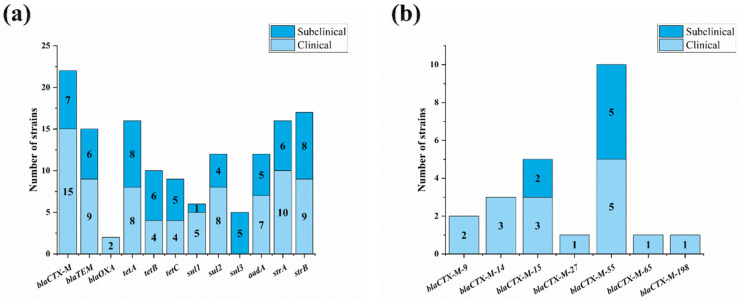
Distribution of resistance genes of mastitis-related *E. coli*. (**a**) Distribution of common drug-resistant genes of four main classes of antibiotic among 87 *E. coli* strains; (**b**) subtypes of *blaCTX-M* genes in mastitis strains.

To further investigate the mechanisms of four classes of compounds with serious bacterial resistance, 12 common antibiotic resistance genes were found in the mastitis-related *E. coli* strains (Supplementary Figure S3). Three *β*-lactam resistance genes were found in the isolates. *BlaCTX-M* and *blaTEM* took relatively high percentages, with detection rates of 33.3% (22/87) and 24.1% (15/87), respectively. *BlaOXA* was only detected in two isolates (2.3%). For tetracycline resistance genes, the detection rates of *tetA*, *tetB* and *tetC* were 27.6% (16/87), 18.4% (10/87) and 16.1% (9/87), respectively. For sulfonamide resistance genes, the detection rates of *sul1*, *sul2* and *sul3* were 8.0% (6/87), 18.4% (12/87) and 11.5% (5/87), respectively. For aminoglycoside resistance genes, the detection rates of *aadA*, *strA* and *strB* were 19.5% (12/87), 25.3% (16/87) and 28.7% (17/87), respectively (Figure 4a).

The results of the difference analysis of resistance genes showed that there was only a significant difference in the distribution of *blaCTX-M* between the clinical and subclinical isolates (*p* < 0.05). The sequencing results showed that there were seven subtypes of *blaCTX-M* found in mastitis-related *E. coli* isolates, including *blaCTX-M-9*, *blaCTX-M-14*, *blaCTX-M-15*, *blaCTX-M-55*, *blaCTX-M-65*, *blaCTX-M-198* and *blaCTX-M-271*. *blaCTX-M-55* (43.5%, 10/22) was the most prevalent subtype, followed by *blaCTX-M-15* (21.7%, 5/22). Different from *blaCTX-M-55* and *blaCTX-M-15*, *blaCTX-M-9*, *blaCTX-M-14*, *blaCTX-M-65*, *blaCTX-M-198* and *blaCTX-M-271* were only observed in clinical mastitis isolates (Figure 4b).

### 3.6. Phylogenetic Tree of Mastitis Isolates

**Figure 5:**
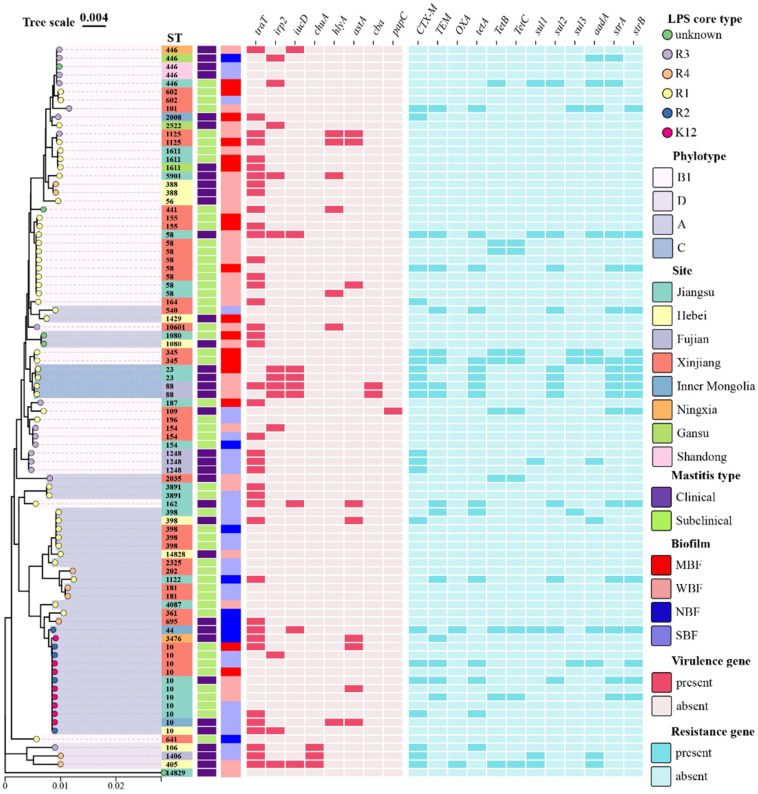
Phylogenetic tree of 87 mastitis-related *E. coli* strains according to seven housekeeping genes (*adk*, *fumC*, *gyrB*, *icd*, *mdh*, *purA*, *recA*). The visualization, annotation and decoration of all trees were carried out utilizing tvBOT (https://www.chiplot.online/tvbot.html, accessed on 22 August 2024). SBF: strain with strong biofilm formation; MBF: strain with moderate biofilm formation; WBF: strain with weak biofilm formation; NBF: strain with no biofilm formation.

The isolates from different provinces showed high heterogeneity, including mastitis types, STs, LPS core types, biofilms, virulence genes and resistance genes. However, R2 and K12 mastitis-related *E. coli* were closely correlated with ST10, while all the ST58 strains belonged to the R1 type (Figure 5).

## 4. Discussion

Both bovine clinical and subclinical mastitis caused by *E. coli* are seen at a high frequency in dairy farming. Most previous studies focused on clinical mastitis-related strains. Here, we mainly paid attention to the differences between clinical and subclinical mastitis-related *E. coli* isolates; several differences were found between the two groups, including virulence genes, biofilms and antimicrobial susceptibility.

Virulence genes play an important role in the pathogenesis of *E. coli*, as the harmless common *E. coli* that inhabit intestines could convert into pathogens when they acquire virulence genes. In this study, most extraintestinal pathogenic *E. coli* (ExPEC) virulence genes were also absent in the detected mastitis-related *E. coli* strains [19]. However, frequently reported genes in mastitis-related *E. coli* (*traT*, *irp2* and *iucD*) were more common in clinical mastitis-related *E. coli* isolates than in subclinical mastitis isolates [20]. The presence of serum resistance factor, TraT reduced the sensitivity of *E. coli* cells to phagocytosis by macrophages. Generally, *traT* is associated with IncF plasmids [21]. The *irp2* gene is linked to the synthesis and regulation of siderophores. Typically, it is part of the high-pathogenicity island, which is located in the chromosomes of pathogenic *E. coli* strains [22]. Thus, the mastitis-related *E. coli* strains containing *irp2* should be examined in clinical mastitis as they might cause serious clinical symptoms in the udders of dairy cows. The *iucD* gene encodes an enzyme involved in the biosynthesis of the siderophore aerobactin, which aids in iron acquisition. It is typically found in the ColV plasmid associated with pathogenic strains [23]. The presence of these genes might be related to the severity of bovine mastitis, and the immune response caused by subclinical mastitis was usually milder than that of acute mastitis [24].

Biofilms are also considered to be an important pathogenic factor for bacteria, and many mastitis pathogens have been reported to have the ability to form biofilms, including *E. coli* [25,26]. It helps bacteria to evade the host’s innate immune system, increasing resistance to antimicrobial agents. Biofilms are responsible for chronic infection or persistent infection by many pathogenic bacteria. In our study, we observed stronger biofilm formation abilities in subclinical isolates than in clinical strains. It is possible that some subclinical mastitis-related *E. coli* isolates survive in the mammary gland for a lengthy period, inducing persistent infection with the help of strong biofilm formation abilities. Meanwhile, the immune responses in the udders of cows with subclinical mastitis are mild, which is conducive to the persistent infection of subclinical mastitis-related *E. coli* [27].

Antimicrobial resistance (AMR) is a major concern for animal bacterial diseases. In this study, polymyxin B and tetracycline were the most and the least susceptible agents for mastitis-related *E. coli*, respectively, and more than a third of the strains were identified as MDR strains. However, it did not seem to be as serious as other animal-origin *E. coli* isolates [28,29]. In fact, this might account for the tight restrictions on antibiotic use in mastitis. Broad-spectrum cephalosporins and fluoroquinolones are only recommended for severe clinical symptoms [30,31]. For antibiotic resistance, the resistance to ampicillin of *E. coli* strains was the only difference between the clinical and subclinical mastitis-related *E. coli* strains. We speculate that this resulted from the induction of the widespread use of penicillin for the treatment of clinical mastitis, while subclinical mastitis lacked timely diagnosis and treatment. The further determination of resistance genes supported our susceptibility results, and it was possible that the distribution of *blaCTX-M* led to the difference between clinical and subclinical mastitis-related *E. coli*.

Despite a relatively strict medication strategy, quite a few isolates exhibited multiple-drug resistance, and many resistance genes were found in them. Among these multiple-drug resistance strains, extended-spectrum beta-lactamase-producing *E. coli* (ESBL-Ec) are a major concern, as they could be resistant to a broad range of clinically commonly used antibiotics, increasing the risk of treatment failure. This is an urgent global health issue both in human and veterinary medicine. CTX-M-family ESBLs have served as the dominating contributor to the multidrug-resistant profile of *E. coli* over the past decade, and many of them were frequently associated with insertion sequence (IS) ISEcp1, which could transfer horizontally to other bacteria [9,32]. It was notable that the most popular *blaCTX-M* subtype was *blaCTX-M-55*, which replaced the most prevalent subtype *blaCTX-M-15* in mastitis-related *E. coli* [33]. This was identified with the potential risk of the rapid spread of *blaCTX-M-55-positive E. coli* all over the world [34]. *BlaOXA* is another important resistance gene in *E. coli* as it is closely related to carbapenem resistance, which is critical for both human and veterinary medicine. Overall, these results remind us of the need to enhance the long-term continuous surveillance of ESBL-positive *E. coli* in bovine mastitis.

Molecular subtyping is beneficial for tracing back to sources and understanding transmission pathways. In general, the details of the MLST phylogenetic tree of 87 mastitis-related *E. coli* strains showed considerable genetic diversity, and it was hard to distinguish clinical and subclinical mastitis-related *E. coli* as both exhibited high-heterogeneity phylogroups, LPS core types and STs. However, some patterns of resistant genes or virulence genes were observed in the same STs, such as ST23 and ST88. It would be useful to further explore the epidemiological and clinical implications, especially regarding inter-species transmission and the risks associated with resistant strains.

## 5. Conclusions

In summary, the present results showed that mastitis-related *E. coli* strains had a high heterogeneity and no significant differences in phylogroups, LPS core types and STs, while virulence genes, biofilm formation, antimicrobial susceptibility and resistance genes were different between the clinical and subclinical group strains. This study aims to provide references for the study of the pathogenic mechanisms of *E. coli* isolates from different types of mastitis.

## Figures and Tables

**Table 1:** Antimicrobial susceptibility profiles of *E. coli* isolates.

Antibiotic Class	Drug Agents	Drug Breakpoint (R S I, mm)	Percentage of Susceptible Strains (S)	Percentage of Intermediate Strains (I)	Percentage of Resistant Strains (R)
β-Lactams	Ampicillin	≤13 14~16 ≥17	49.4% (43/87)	13.8% (12/87)	36.8% (32/87)
	Amoxicillin/clavulanic acid	≤13 14~17 ≥18	96.6% (84/87)	2.3% (2/87)	1.1% (1/87)
	Cefepime	≤14 15~17 ≥18	89.7% (79/87)	4.6% (4/87)	5.7% (4/87)
	Ceftazidime	≤14 15~17 ≥18	86.2% (75/87)	6.9% (6/87)	6.9% (6/87)
Sulfonamides	Cotrimoxazole	≤10 11~15 ≥16	69.0% (60/87)	2.3% (2/87)	28.7% (25/87)
Tetracyclines	Tetracycline	≤14 15~18 ≥19	41.4% (36/87)	20.7% (18/87)	37.9% (33/87)
Phenicols	Florfenicol	≤12 13~17 ≥18	87.4% (76/87)	0.0% (0/87)	12.6% (11/87)
Aminoglycosides	Kanamycin	≤13 14~17 ≥18	69.0% (60/87)	16.1% (14/87)	14.9% (13/87)
	Gentamycin	≤12 13~14 ≥15	79.3% (69/87)	3.4% (3/87)	17.2% (15/87)
	Streptomycin	≤14 15~18 ≥19	50.6% (44/87)	14.9% (13/87)	34.5% (30/87)
Fluoroquinolones	Enrofloxacin	≤14 15~20 ≥21	93.1% (81/87)	2.3% (2/87)	4.6% (4/87)
Polymyxin	Polymyxin B	≤8 9~11 ≥12	97.7% (85/87)	2.3% (2/87)	1.1% (0/87)

**Table 2:** Distribution of MDR strains in mastitis-related *E. coli*.

Classes of Resistant Antibiotics	Number of Strains of Mastitis-Related *E. coli*	Percentage of Mastitis-Related *E. coli*	Percentage of Clinical Strains	Percentage of Subclinical Strains
0	32	36.8%	36.1%	37.3%
1	19	21.8%	13.9%	27.5%
2	10	11.5%	16.7%	7.8%
3	10	11.5%	13.9%	9.8%
4	7	8.0%	8.3%	7.8%
5	6	6.9%	8.3%	5.9%
6	3	3.4%	2.8%	3.9%

## Data Availability

The datasets generated during the current study are available from the corresponding author on reasonable request.

## Supplementary Materials

The following supporting information can be downloaded at https://www.mdpi.com/article/10.3390/vetsci12020132/s1, Figure S1: PCR amplification of phylo-typing genes referred to Clermont’s PCR methods; Figure S2: PCR amplification of virulence genes existing in mastitis *E. coli* strains; Figure S3: PCR amplification of common antibiotic-resistant genes of mastitis *E. coli* strains; Table S1: specific information of 131 isolates of *E. coli*; Table S2: primers used in this study; Table S3: MLST of mastitis-associated *E. coli* isolates.

## Abbreviations

CMT	California Mastitis Test
MLST	Multilocus sequence typing
LPS	Lipopolysaccharide
ExPEC	Extraintestinal pathogenic *E. coli*
AMR	Antimicrobial resistance
MDR	Multiple-drug resistance
ESBL-Ec	Extended-spectrum beta-lactamase-producing *E. coli*
