# Supplementary material for: Difference Analysis on Virulence Genes, Biofilms and Antimicrobial Susceptibility of Escherichia coli from Clinical and Subclinical Bovine Mastitis
Source: Vet Sci. 2025 Feb 6;12(2):132. doi: 10.3390/vetsci12020132 (PMC11861582; doi:10.3390/vetsci12020132)
Supplement: Supplementary file 1 [file vetsci-12-00132-s001.zip › supplementary figures.pdf]

Supplementary Figure S1

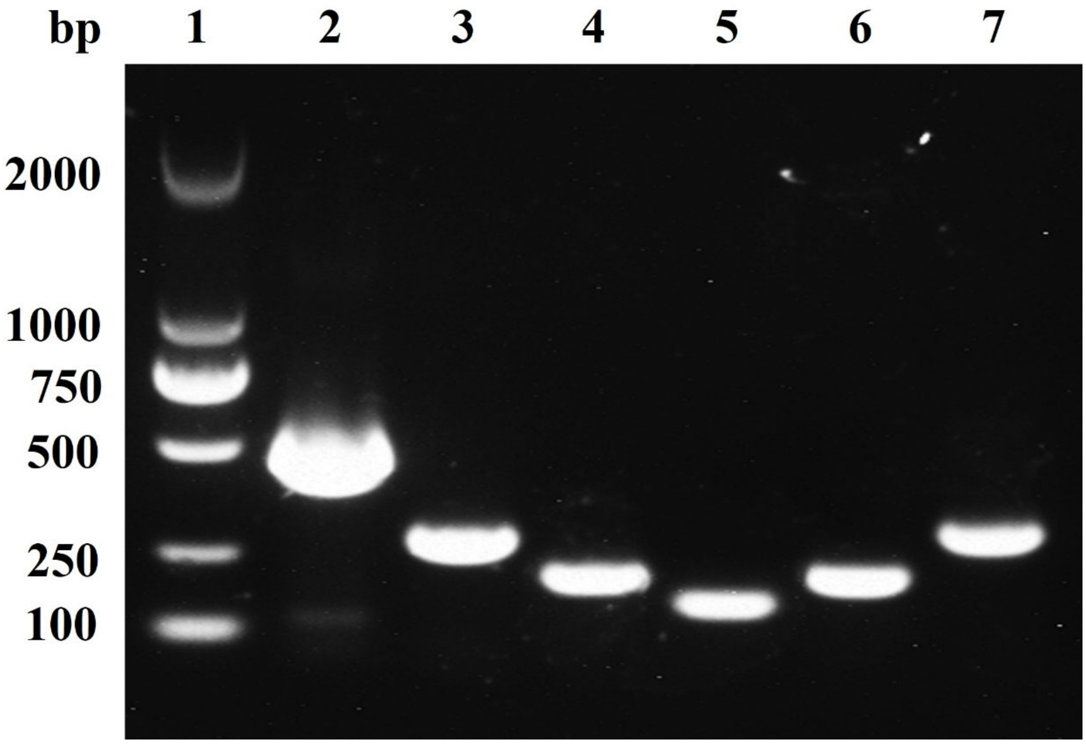

Supplementary Figure S1. PCR amplification of phylo-typing genes referred to Clermont's PCR methods. line 1: DNA Maker DL2000; line 2: *arpA*; line 3: *chuA*; line 4: *yjaA*; line 5: *TspE4.C2*; line 6: *trpAgp*C; line 7: ArpAgpE.

Supplement Figure S2

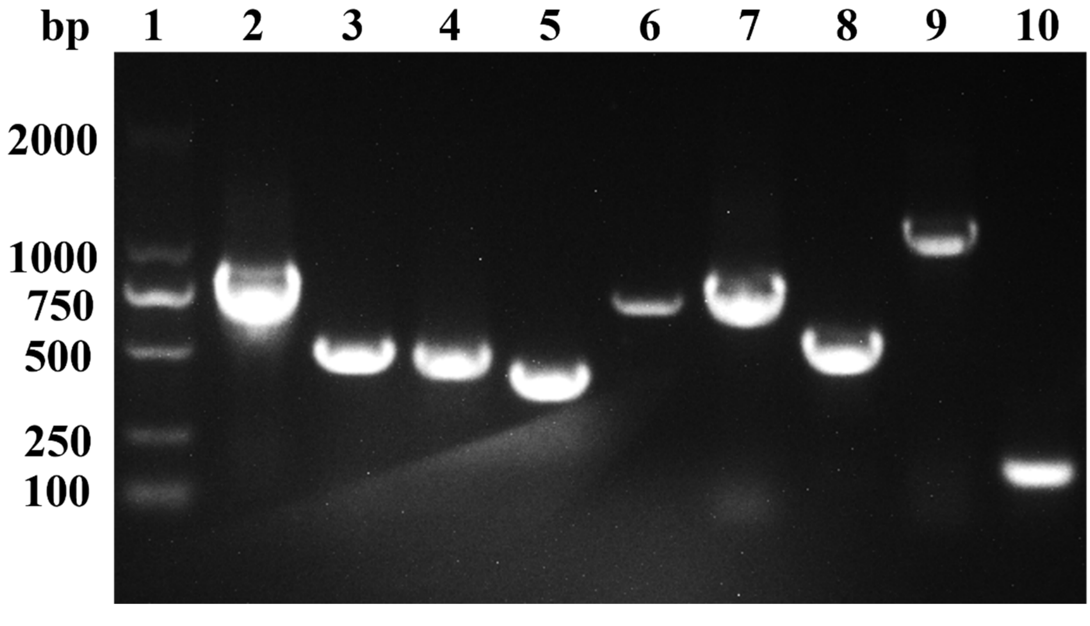

Supplement Figure S2. PCR amplification of virulence genes existing in mastitis *E. coli* strains. line 1: DNA maker: DL2000; line 2: *aer*; line 3: *ompA*; line 4: *traT*; line 5: *irp2*; line 6: *iucD*; line 7: *cba*; line 8: *hlyA*; line 9: *papC*; line 10: *east*1.

Supplement Figure S3

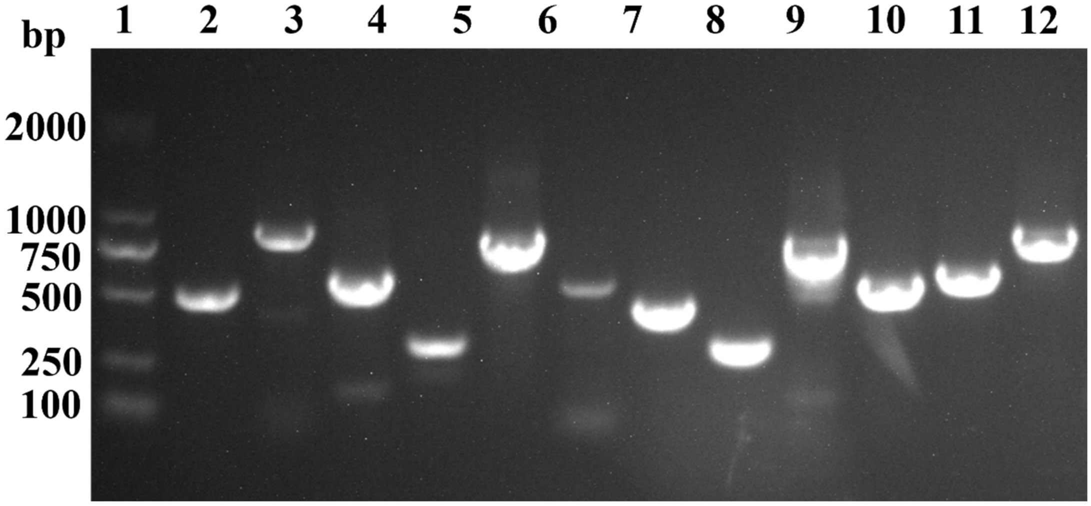

Supplement Figure S3. PCR amplification of common antibiotic-resistant genes of mastitis *E. coli* strains. line 1: DNA maker: DL2000; line 2: *blaTEM*; line 3 *blaCTX-M*; line 4: *blaOXA*; line 5: *tetA*; line 6: *tetB*; line 7: *tetC*; line 8: *sul1*; line 9: *sul2*; line 10: *sul3*; line 11: *aadA1*; line 12: *strA*; line 13: *strB*;
