# Supplementary material for: Difference Analysis on Virulence Genes, Biofilms and Antimicrobial Susceptibility of Escherichia coli from Clinical and Subclinical Bovine Mastitis
Source: Vet Sci. 2025 Feb 6;12(2):132. doi: 10.3390/vetsci12020132 (PMC11861582; doi:10.3390/vetsci12020132)
Supplement: Supplementary file 1 [file vetsci-12-00132-s001.zip › supplementary tables.pdf]

Supplementary Table S1. Specific information of 131 isolates of *E. coli*

Strains	Separation sites	Separation times	Mastitis types
A1	Suqian, Jiangsu	2020.8	Subclinical
A9	Suqian, Jiangsu	2020.8	Subclinical
A11	Suqian, Jiangsu	2020.8	Subclinical
A12	Suqian, Jiangsu	2020.8	Subclinical
A16	Suqian, Jiangsu	2020.8	Subclinical
A26	Suqian, Jiangsu	2020.8	Subclinical
A31	Suqian, Jiangsu	2020.8	Subclinical
A33	Suqian, Jiangsu	2020.8	Subclinical
B7	Suqian, Jiangsu	2020.8	Subclinical
B44	Suqian, Jiangsu	2020.8	Subclinical
B46	Suqian, Jiangsu	2020.8	Subclinical
C6	Xuzhou, Jiangsu	2020.8	Subclinical
C13	Xuzhou, Jiangsu	2020.8	Subclinical
C27	Xuzhou, Jiangsu	2020.8	Subclinical
C33	Xuzhou, Jiangsu	2020.8	Subclinical
C43	Xuzhou, Jiangsu	2020.8	Subclinical
C46	Xuzhou, Jiangsu	2020.8	Subclinical
1XJ_102	Alaer, Xinjiang	2020.9	Subclinical
1XJ_112	Alaer, Xinjiang	2020.9	Subclinical
1XJ_123	Alaer, Xinjiang	2020.9	Subclinical
1XJ_127	Alaer, Xinjiang	2020.9	Subclinical
1XJ_137	Alaer, Xinjiang	2020.9	Subclinical
1XJ_150	Alaer, Xinjiang	2020.9	Subclinical
1XJ_159	Alaer, Xinjiang	2020.9	Subclinical
1XJ_172	Alaer, Xinjiang	2020.9	Subclinical
1XJ_191	Alaer, Xinjiang	2020.9	Subclinical
2XJ_6	Alaer, Xinjiang	2021.3	Subclinical
2XJ_7	Alaer, Xinjiang	2021.3	Subclinical
2XJ_8	Alaer, Xinjiang	2021.3	Subclinical
2XJ_9	Alaer, Xinjiang	2021.3	Subclinical
2XJ_13	Alaer, Xinjiang	2021.3	Subclinical
3XJ_32	Alaer, Xinjiang	2021.3	Subclinical
3XJ_33	Alaer, Xinjiang	2021.3	Subclinical
3XJ_34	Alaer, Xinjiang	2021.3	Subclinical
3XJ_35	Alaer, Xinjiang	2021.3	Subclinical
3XJ_36	Alaer, Xinjiang	2021.3	Subclinical
3XJ_37	Alaer, Xinjiang	2021.3	Subclinical
3XJ_38	Alaer, Xinjiang	2021.3	Subclinical
3XJ_39	Alaer, Xinjiang	2021.3	Subclinical

3XJ_40	Alaer, Xinjiang	2021.3	Subclinical
3XJ_41	Alaer, Xinjiang	2021.3	Subclinical
3XJ_42	Alaer, Xinjiang	2021.3	Subclinical
3XJ_43	Alaer, Xinjiang	2021.3	Subclinical
3XJ_44	Alaer, Xinjiang	2021.3	Subclinical
3XJ_45	Alaer, Xinjiang	2021.3	Subclinical
3XJ_46	Alaer, Xinjiang	2021.3	Subclinical
3XJ_47	Alaer, Xinjiang	2021.3	Subclinical
3XJ_48	Alaer, Xinjiang	2021.3	Subclinical
3XJ_49	Alaer, Xinjiang	2021.3	Subclinical
3XJ_50	Alaer, Xinjiang	2021.3	Subclinical
3XJ_51	Alaer, Xinjiang	2021.3	Subclinical
B1	Suqian, Jiangsu	2020.8	Clinical
B3	Suqian, Jiangsu	2020.8	Clinical
B4	Suqian, Jiangsu	2020.8	Clinical
B5	Suqian, Jiangsu	2020.8	Clinical
H88	Xuzhou, Jiangsu	2019.12	Clinical
H118	Nanjing, Jiangsu	2020.4	Clinical
H119	Nanjing, Jiangsu	2020.4	Clinical
H179	Liaocheng, Shandong	2020.4	Clinical
H180	Liaocheng, Shandong	2020.4	Clinical
J40	Zhangjiakou, Hebei	2020.6	Clinical
J41	Zhangjiakou, Hebei	2020.6	Clinical
J49	Zhangjiakou, Hebei	2020.6	Clinical
J57	Zhangjiakou, Hebei	2020.6	Clinical
J82	Zhangjiakou, Hebei	2020.6	Clinical
J90	Zhangjiakou, Hebei	2020.6	Clinical
JL53	Hengshui, Hebei	2020.7	Clinical
JL60	Langfang, Hebei	2020.7	Clinical
JL63	Langfang, Hebei	2020.7	Clinical
JL68	Langfang, Hebei	2020.7	Clinical
J130	Yinchuan, Ningxia	2020.6	Clinical
JL106	Wuzhong, Ningxia	2020.7	Clinical
H136	Inner Mongolia	2020.4	Clinical
H170	Inner Mongolia	2020.4	Clinical
H184	Inner Mongolia	2020.4	Clinical
J162	Linxia, Gansu	2020.6	Clinical
JL65	Linxia, Gansu	2020.6	Clinical
JL14	Linxia, Gansu	2020.7	Clinical
FJ_1	Nanping, Fujian	2020.10	Clinical
FJ_4	Nanping, Fujian	2020.10	Clinical
FJ_8	Nanping, Fujian	2020.10	Clinical

FJ_10	Nanping, Fujian	2020.10	Clinical
FJ_11	Nanping, Fujian	2020.10	Clinical
FJ_15	Nanping, Fujian	2020.10	Clinical
XJ_2	Ili, Xinjiang	2020.10	Clinical
XJ_3	Ili, Xinjiang	2020.10	Clinical
XJ_4	Ili, Xinjiang	2020.10	Clinical

Supplementary Table S2. Primers used in this study

Class	Primers	Oligonucleotide sequence (5'→3')	Descriptions	Product size（bp）
Bacteria identification	16S-F	ACGCGTCGACAGAGTTTGATCCTGGCT	Identifying of *E.coli*	1466
	16S-R	CGCGGATCCGCTACCTTGTTACGACTT		
Quadruplex PCR method for phylogroup assignment	chuA.2-F	ATGGTACCGGACGAACCAAC	Determination of *Escherichia coli* phylogenetic group by quadruplex PCR method	288
	chuA.2-R	TGCCGCCAGTACCAAAGACA		
	TspE4C2.2-F	CACTATTCGTAAGGTCATCC		152
	TspE4C2.2-R	AGTTTATCGCTGCGGGTCGC		
	yiaA.2-F	CAAACGTGAAGTGTCAGGAG		211
	yiaA.2-R	AATGCGTTCCTCAACCTGTG		
	arpA.2-F	AACGCTATTCGCCAGCTTGC		400
	arpA.2-R	TCTCCCCATACCGTACGCTA		
	arpAgpE1	GATTCCATCTTGTCAAAATATGCC		301
	arpAgpE2	GAAAAGAAAAAGAATTCCCAAGAG		
	trpAgpC1	AGTTTTATGCCCAGTGCGAG		219
	trpAgpC2	TCTGCGCCGGTCACGCCC		
Virulence genes	afaE-F	CTGCGCTTTGAGGAAGAGGAG	Adhesion related genes	414
	afaE-R	GCAAGTGGTGATAACGCTGATAC		
	eaeA-F	CGGCGGCCAGGTTAATTTCT		713
	eaeA-R	CGTAGCTGTTAACCACACCG		
	papC-F	TGGTTGGGTCAGCAATCGT		1185
	papC-R	GCCAACTTTTGCAGCAGCTAAATAT		

saa-F	GTAAACAGCGGCTTCAGCAC		1230
saa-R	GCCAACTAAAACGTCAGCCC		
sfa-F	TGGTCACGGATTATGTAACGGT		297
sfa-R	ACGCTATGACAGAACCTGGT		
irp2-F	CTGATGAACTCACTCGCTATCC	Siderophore related genes	440
irp2-R	AGCATCTCCTGGCTCTGCTC		
aer-F	CTGCCTTCATTACCGCTTCG		850
aer-R	CCTGTGCCACAATATCTTCCAT		
iucD-F	GGGCCATTTAATCTCAGCAT		743
iucD-R	GATGTCATTTTCTGCTCATC		
escV-F	GCGTGTTGCTGAAGTGAGTG	Type III Secretion System (T3SS) genes	1033
escV-R	TTGCCAGTTCCAAATGTTCT		
sepD-F	ATGATTGTGACTGGCTAACG		585
sepD-R	TTGGCAAAGATACCACTTCT		
east1-F	GGCTATCGATGAACGATATC	Toxin	180
east1-R	CTTTCAGGTCGCGAGTGAC		
stx2e-F	AATCATACACCACCAGGAAG		671
stx2e-R	TCGCCAGTTATCTGACATTC		
CNF1-F	TGCCGTGGTTTAGTCGGATTG		456
CNF1-R	TGCGATTTAAGGCGTTCATG		
cba-F	AAGAACTTGAAGAGGGAGACT		774
cba-R	CACCGACACTAATGATGACC		
hlyA-F	TAACTTAGGAAAGGCAGGCGG		573
hlyA-R	TCGGCAATGGACAGGAATGAG		

	estB-F	CGATCATCCCACTATTCTTC		543
	estB-R	GTCTGTATTGCTTCGACCTG		
	ompA-F	ACAACAACGCGGGCATTTAC	Invasion factor	513
	ompA-R	TCCTGCATCTTTCGGCCAAT		
	traT-F	GCACAGCAATCAAGAAGCGTAA	Serum resistance gene	474
	traT-R	CCGTTGTCACCGTTGCCTTA		
Resistance genes	tetA-F	TGAAACCCAACAGACCCCTG	Common tetracycline antibiotic resistance genes	315
	tetA-R	CCACGATCCGCCCGATATAG		
	tetB-F	GCCGATACCACCTCAGC		794
	tetB-R	ATCCAGCCATCCCAAAT		
	tetC-F	TAACAAATGGTTGGTCT		347
	tetC-R	TTTATCTTTCAAGCTCA		
	CTX-M-F	ACTTCAGCCACACGGATTCA	Common β-lactam antibiotic resistance genes	905
	CTX-M-R	AAGTGGAGCGACAGAGC		
	SHV-F	CTCAGGGCTAGAAGTGTCAT		333
	SHV-R	CACTAACCCAGAAAGTCGAG		
	TEM-F	CAGCGGTAAGATCCTTGAGA		541
	TEM-R	AGCAATAAACCAGCCAGCCG		
	KPC-F	CTCTGGTGCGGCAGAAGTAC		791
	KPC-R	TGGCCGTCGACGATAAACTC		
	OXA-F	CTTCAGCAGATAAATGACGGGGTAG		555
	OXA-R	GTGGGTAAACCGCCATATTTTCC		
	sul1-F	CGGCGTGGGCTACCTGAACG	Common sulfonamides resistance genes	433
	sul1-R	GCCGATCGCGTGAAGTTCCG		
	sul2-F	GCGCTCAAGGCAGATGGCATT		285

	sul2-R	GCGTTTGATACCGGCACCCGT		
	sul3-F	GAGCAAGATTTTTGGAATCG		790
	sul3-R	CTAACCTAGGGCTTTGGATAT		
	aadA-F	GTGGATGGCGGCCTGAAGCC	Common aminoglycosides resistance genes	527
	aadA-R	ATTGCCCAGTCGGCAGCG		
	strA-F	CATTCTGACTGGTTGCCTG		564
	strA-R	GCATTGAAGAGTTTTAGGGTC		
	strB-F	TGCTCATTGGCACGTCGCA		803
	strB-R	TCTGTCGCACCTGCTTGATC		

Supplementary Table S3. MLST of mastitis-associated *E. coli* isolates

Strains	Sequence type	***Adk*** ***536***	***fumC*** ***469***	***gyrB*** ***460***	***Icd*** ***518***	***Mdh*** ***452***	***purA*** ***478***	***recA*** ***510***
A1	58	6	4	4	16	24	8	14
A9	187	6	69	4	16	9	13	7
A11	4087	10	11	57	8	7	8	6
A12	398	64	7	1	1	8	8	6
A16	58	6	4	4	16	24	8	14
A26	3891	9	8	12	138	9	12	6
A31	446	6	19	3	26	11	8	6
A33	3891	9	8	12	138	9	12	6
B1	23	6	4	12	1	20	13	7
B3	23	6	4	12	1	20	13	7
B4	58	6	4	4	16	24	8	14
B5	10	10	11	4	8	8	8	2
B7	1122	8	11	57	1	7	18	6
B44	1611	6	4	15	18	9	26	7
B46	1611	6	4	15	18	9	26	7
C6	1080	6	4	7	9	7	7	56
C13	10	10	11	4	8	8	8	2
C27	154	6	6	5	10	9	8	6
C33	10	10	11	4	8	8	8	2
C43	10	10	11	4	8	8	8	2
C46	10	10	11	4	8	8	8	2
XJ_2	109	6	6	1	16	9	13	2
XJ_3	695	10	11	4	12	8	18	2
XJ_4	2035	6	8	4	225	9	23	7
1XJ_102	602	6	19	33	26	11	8	6
1XJ_112	10601	6	6	3	10	7	18	7
1XJ_123	154	6	6	5	10	9	8	6
1XJ_127	10	10	11	4	8	8	8	2
1XJ_137	602	6	19	33	26	11	8	6
1XJ_150	2325	8	7	4	8	8	18	6
1XJ_159	196	6	19	3	16	9	8	6
1XJ_172	154	6	6	5	10	9	8	6
1XJ_191	155	6	4	14	16	24	8	14
2XJ_6	58	6	4	4	16	24	8	14
2XJ_7	58	6	4	4	16	24	8	14
2XJ_8	1125	6	4	15	18	24	26	7

2XJ_9	1125	6	4	15	18	24	26	7
2XJ_13	164	6	4	32	16	12	8	7
3XJ_32	345	6	4	14	1	20	62	7
3XJ_33	58	6	4	4	16	24	8	14
3XJ_34	202	64	11	5	8	8	8	2
3XJ_35	361	10	99	5	91	8	7	2
3XJ_36	58	6	4	4	16	24	8	14
3XJ_37	540	6	7	57	1	8	8	2
3XJ_38	398	64	7	1	1	8	8	6
3XJ_39	155	6	4	14	16	24	8	14
3XJ_40	10	10	11	4	8	8	8	2
3XJ_41	58	6	4	4	16	24	8	14
3XJ_42	641	9	6	33	131	24	8	7
3XJ_43	181	8	11	4	8	7	8	6
3XJ_44	10	10	11	4	8	8	8	2
3XJ_45	10	10	11	4	8	8	8	2
3XJ_46	101	43	41	15	18	11	7	6
3XJ_47	181	8	11	4	8	7	8	6
3XJ_48	345	6	4	14	1	20	62	7
3XJ_49	398	64	7	1	1	8	8	6
3XJ_50	398	64	7	1	1	8	8	6
3XJ_51	441	6	4	15	103	24	8	7
FJ_1	1406	46	156	2	25	5	16	19
FJ_4	1248	6	29	12	1	9	8	7
FJ_8	88	6	4	12	1	20	12	7
FJ_10	88	6	4	12	1	20	12	7
FJ_11	1248	6	29	12	1	9	8	7
FJ_15	1248	6	29	12	1	9	8	7
H88	5901	6	763	14	18	7	7	71
H118	162	9	65	5	1	9	13	6
H119	14829	356	1998	1483	699	349	322	509
H136	2008	6	6	5	136	11	8	6
H170	10	10	11	4	8	8	8	2
H179	446	6	19	3	26	11	8	6
H181	446	6	19	3	26	11	8	6
H184	44	10	11	4	8	8	8	7
J40	405	35	37	29	25	4	5	73
J41	1429	6	4	109	1	8	8	6
J49	106	21	35	27	6	5	8	4

J57	14828	8	7	1	1869	8	7	6
J82	1080	6	4	7	9	7	7	56
J90	398	64	7	1	1	8	8	6
J130	446	6	19	3	26	11	8	6
J162	446	6	19	3	26	11	8	6
J165	1611	6	4	15	18	9	26	7
JL14	2522	6	29	5	18	11	8	2
JL53	388	6	4	12	18	24	7	6
JL60	388	6	4	12	18	24	7	6
JL63	10	10	11	4	8	8	8	2
JL68	56	6	4	4	18	24	5	14
JL106	3476	10	11	4	8	291	8	2
